# Experimental Research on the Performance of Recycled Waste Concrete Powder (RWCP) on Concrete

**DOI:** 10.3390/ma17215319

**Published:** 2024-10-31

**Authors:** Shuai Wang, Aixun Wang, Xudong Fu, Xianwei Zhang, Zhe Li, Yongjun Guo, Shenghao Li, Mingzhao Wang

**Affiliations:** 1 Wuhan Construction Group Co., Ltd., Wuhan 430056, China; wangshuai@wceg.com.cn (S.W.);; 2 School of Civil Engineering and Architecture, Wuhan University, Wuhan 430072, China; xdfu@whu.edu.cn; 3 Research Center on Environmental Technology of Marine Engineering, China Institute of Ocean Engineering (Tsingtao), Qingdao 266555, China; 4 School of Environmental and Municipal Engineering, Qingdao University of Technology, Qingdao 266520, China; 5 School of Materials Science and Engineering, Wuhan University of Science and Technology, Wuhan 430071, China; lishenghao@wust.edu.cn; 6 School of Automotive and Traffic Engineering, Wuhan University of Science and Technology, Wuhan 430071, China

**Keywords:** recycled waste concrete powder, activity index, mechanical property, working property, penetration resistance

## Abstract

Waste concrete is a large amount of solid waste produced in the process of urban construction and renewal in China. Its resource utilization is of great significance for saving mineral resources and improving urban environmental quality. The present study was designed to investigate the effects of mechanical grinding time on the particle size distribution and activity of recycled waste concrete powder (RWCP). Combined with unconfined compressive strength, slump, electric flux and chloride ion penetration resistance tests, the effects of RWCP on the mechanical properties, working performance and impermeability of concrete were analyzed, and the phase and microstructure of concrete containing RWCP were analyzed by XRD and SEM. The results showed that the RWCP is mainly composed of quartz, gismondine, C_2_S, cancrinite and portlandite. The optimum activity of RWCP obtained by ball milling for 45 min was 44.41%. RWCP can improve the fluidity of concrete and shorten the initial setting time of concrete. When the blast furnace slag in the concrete was replaced by the RWCP, the early strength and impermeability of the concrete decreased. When RWCP replaced blast furnace slag by 69.1%, the UCS of the concrete at 1, 3, 7, and 14 d decreased from 9.56, 22.1, 34.1, and 41.2 MPa to 5.9, 14.5, 22.7, and 33.2 MPa, respectively. While RWCP replaced fly ash, the normal strength of concrete increased with the increase in fly ash replacement amount. When RWCP completely replaced FA in concrete, the 28-day strength of the concrete increased from 45.2 MPa to 50.8 MPa. The impermeability results showed that the appropriate substitution of RWCP for fly ash was beneficial to increase the impermeability of concrete while excessive substitution reduced. Based on these results, the RWCP has the potential for large-scale application in the preparation of concrete.

## 1. Introduction

Recent years have witnessed accelerated urban construction and upgrading in China. As a result, increasing attention has been paid to the question of how to deal with the construction waste arising from demolition and reconstruction activities in an effective and environmentally friendly way, such as waste concrete (WC), demolition waste, decoration waste, engineering residues and slurries, etc., to reduce environmental harm and impact [1,2,3]. China’s total construction waste stock has reached 20 billion tons, and it will continue to generate billions of tons per year [4,5]. It is expected that the construction waste will keep growing rapidly in the future decade [6]. China produces around 50 million tons of WC each year. As WC contributes the largest proportion to construction waste, it is prone to causing environmental pollution if not handled properly [7,8,9]. Large in stock and difficult to degrade, it is often used for backfill and prone to causing environmental pollution or cultivated land occupation [10,11]. The accumulation of waste concrete can easily lead to dust pollution, while the chemicals it contains, such as flame retardants and paints, pose a risk of contaminating soil and water [12]. The current recycling rate of WC in China is less than 5%, lower than that in developed countries. Domestic and foreign scholars have conducted corresponding research on the recycling of WC, aiming to reduce the environmental harm of WC through recycling of concrete or reuse of crushed concrete, and have produced a series of research findings.

Until now, the traditional recycling method of WC was mainly based on classification, crushing and screening [9]; the value-added products obtained by the above methods are mainly recycled WC aggregate. However, due to the increase in water absorption and the decrease in mechanical properties of concrete caused by recycled WC aggregate, the replacement rate of recycled WC aggregate instead of natural aggregate is strictly controlled in relevant specifications [13,14,15]. The main reason for this adverse effect is that the recycled WC aggregate contains a large number of fine powders (FP_S_) (<80 μm) produced when aggregates are stripped from WC, which have a large specific surface area, water absorption and low activity, which affects the use of recycled WC aggregates and these FP_S_ [16,17]. Many studies [18,19] have found that adding FPs from recycled WC aggregate will lead to a decline in the working performance and mechanical properties of concrete, which requires the addition of more water-reducing agents in the production process to ensure the quality of concrete. Bordy [20] confirms that replacing cement mortar with FPs results in a decrease in compressive strength and an increase in total porosity. Furthermore, the reactivity of FPs is lower than that of mineral admixtures, such as mineral powder and fly ash [21]. These negative effects limit the use of FPs in concrete and mortar, prompting research efforts to enhance its performance and increase its applicability in cement materials. Currently, advancements have been made in enhancing the performance of recycled powder through methods such as carbonization, microwave treatment, and mechanical activation. However, these techniques face challenges due to their complicated processing methods and high costs, making practical implementation difficult [22,23,24,25,26]. Currently, scholars are primarily concerned with the FPs obtained during the process of crushing WC to produce recycled aggregate and shaping it. These FPs typically exhibit irregular shapes and a dispersed particle size distribution. However, there is a limited amount of research on the application of recycled waste concrete powder (RWCP) derived from crushing and ball milling total WC in concrete.

In this investigation, RWCP was produced through mechanical ball milling of WC and subsequently utilized in the formulation of concrete. The optimal ball milling duration for RWCP was determined via activity testing. The impact of RWCP on the mechanical properties, construction performance, and impermeability of concrete, achieved by substituting traditional mixed materials with RWCP, was thoroughly examined. Furthermore, XRD and SEM techniques were employed to conduct microstructural and phase analyses of RWCP-incorporated concrete. A comprehensive assessment regarding the feasibility of formulating concrete by replacing the conventional mixture with RWCP was conducted.

## 2. Materials and Methods

### 2.1. Raw Materials

The cement was P.O 42.5 ordinary Portland cement produced by Shandong Shanshui Cement Group Co., Ltd. from Qingdao, China; the coarse aggregate, fine sand, blast furnace slag (BFS), and fly ash (FA) were obtained from Xinlong Construction Group Building Material Technology Co., Ltd. from Qingdao, China, and the grinding aid was obtained from Rushan Baixiang Building Material Co., Ltd from Rushan, China. The grinding aid is mainly used to prevent the adhesion of RWCP to the grinding forging ball during grinding, which was prepared by mixing 60% triethanolamine, 30% sodium chloride and 10% sodium lignosulfonate.

### 2.2. Preparation of RWCP

RWCP was formed by ball milling WC after crushing by jaw crusher. SM-500 cement ball mill (made by Zhongke Co., Ltd. from Cangzhou, China) was used to grind the crushed WC for 15, 30, 45 and 60 min, respectively. The ball mill was loaded with 100 kg of grinding media and operated at a rotational speed of 48 rpm. The amount of grinding aid was 1‰ of the quality of WC. The amount of grinding powder in each group was 5 kg. After the grinding was completed, the grinding door of the small mill was unloaded, the screen door was replaced, and the machine was started again for 5 min to screen the RWCP into the drawer below the machine for collection, and finally, the machine was closed. After the RWCP preparation was completed, the bag was sealed, and the label was attached.

### 2.3. Preparation of Concrete Containing RWCP

The best group for the concrete test specimen was selected, controlling the water–binder ratio, slurry-to-bone ratio and the amount of adhesive material of concrete, and the RWCP was used to replace the blast furnace slag (BFS) and fly ash (FA), respectively, according to the ratio in Table 1 for the preparation of concrete. The mechanical properties, working properties and impermeability of recycled micro-powder concrete were investigated. In addition, sand-free and stone paste samples will be prepared according to the mix ratio in Table 1 and molded under the same curing conditions for microscopic characterization.

### 2.4. Activity Test of RWCP

The activity index of RWCP was tested by *Ground granulated blast furnace slag used for cement, mortar and concrete* (Chinese standard GB/T 18046-2017) [27]. The control group was prepared using 450 g of cement, 1350 g of standard sand, and 225 g of water. In contrast, the test group consisted of 225 g of cement, 225 g of RWCP, 1350 g of standard sand, and 225 g of water. The activity index of RWCP was calculated as the ratio of the compressive strength of the test group to that of the control group.

### 2.5. Strength Test

The unconfined compressive strength (UCS) of concrete after curing for 1, 3, 7, 14, and 28 d was tested by using the automatic pressure-testing machine (BC300D, Beijing Constant-stress Science &Technology Co., Ltd., Beijing, China) at a loading rate of 0.2 kN/s [28]. The concrete compressive strength value was derived from the average of three parallel samples, with an error margin of no more than 10%.

### 2.6. Electric Flux Test and RCM

**Figure 1:**
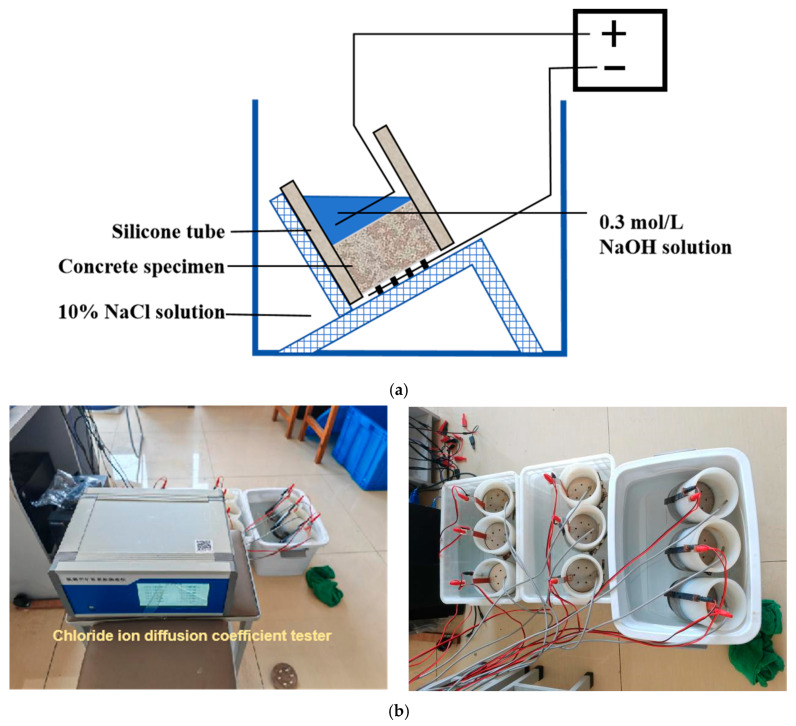
Schematic diagram of chloride ion migration coefficient measurement. (**a**) Water saturation treatment; (**b**) Flowchart of chloride ion measurement.

The electric flux of concrete was tested by the electric flux method in *Long-term Performance and Durability of ordinary concrete* (GB/T50082-2009) [29]. The diffusion coefficient of chloride ions in concrete was determined by the rapid chloride migration method of concrete (RCM) [29]. The RCM method used cylindrical concrete specimens with diameter (100 ± 1) mm and height (50 ± 2) mm. After curing, saturated water treatment was carried out, and then, the specimens were installed in the device as shown in Figure 1. The negative and anode solutions of the specimens were 10% NaCl solution and 0.3 mol/L NaOH solution, respectively. After the test, the specimen was broken into two semi-cylinders along the axis under the pressure testing machine, and 0.1 mol/L of AgNO3 solution color indicator was sprayed on the cross section. After that, the penetration contour of the specimen was drawn with a waterproof pen, the penetration depth was measured, and the chloride ion migration coefficient was calculated.

### 2.7. Micro Characterization

A sample of 3 g passed the 200-mesh sieve, and the Zetium model X-ray fluorescence spectrometer made by Panalytical Company of the Netherlands was used to test the sample by the tablet method.

The sample of 1 g passed through the 200-mesh screen was adopted by the German D8 Advance X-ray diffractometer. A copper target was used for measurement at a scanning Angle of 10°~75° and a scanning speed of 5°/min. Through the diffraction pattern, the composition, internal molecular structure and morphology of the material were analyzed.

The particle size distribution of RWCP was analyzed by a dry test with Malvern 2000 laser particle size analyzer (made by Malvern Panalytical Ltd from Malvern, England).

The microstructure morphology was observed using Hitachi SU8020 scanning electron microscope (SEM) (made by Hitachi Ltd. from Tokyo, Japan).

## 3. Results and Discussion

### 3.1. Characteristics of RWCP

**Figure 2:**
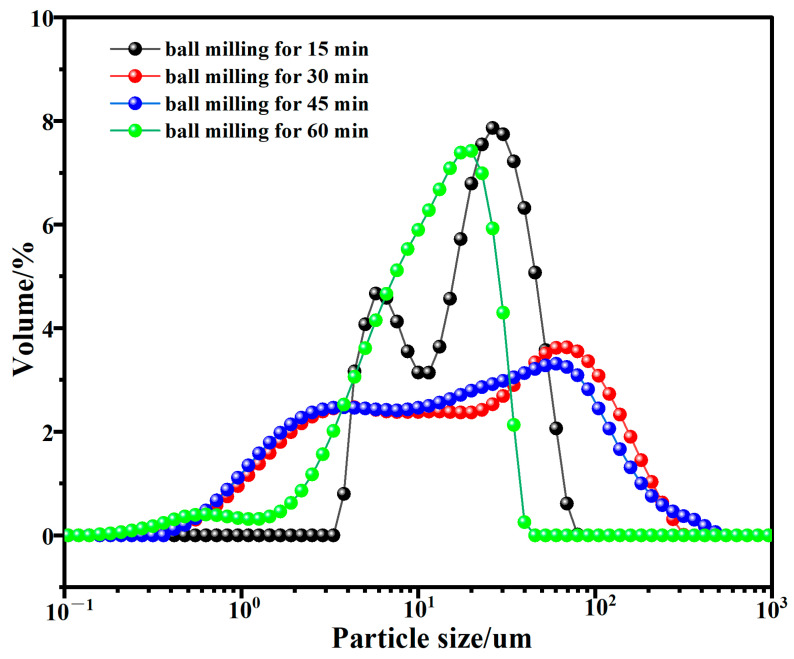
The particle size distribution of RWCP with different ball milling time.

**Figure 3:**
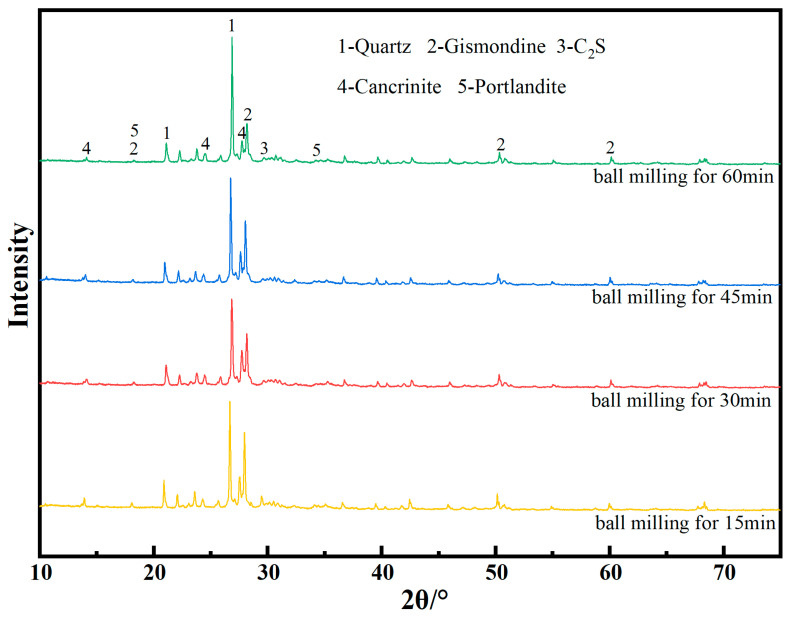
XRD pattern of RWCP prepared by different grinding time.

Figure 2 shows the particle size distribution of the RWCP after different ball milling time. The specific surface area of the RWCP after ball milling for 15, 30, 45 and 60 min are 0.42, 1.5, 1.12 and 1.47 m^2^/g, respectively. The results showed that the specific surface area of RWCP decreases with the increase in grinding time. The XRD analysis (Figure 3) showed that the phase composition of the RWCP prepared with different ball milling times was basically the same, mainly including quartz, gismondine, C_2_S, cancrinite and portlandite.

Table 2 shows the distribution of chemical elements in RWCP (group C). The results showed that the content of SiO_2_ in the RWCP was the highest, reaching 52.46%; the content of CaO, Al_2_O_3_ and Fe_2_O_3_ was 11.436%, 12.998% and 3.077%, respectively; the cumulative content of Si, Ca, Al and Fe in the RWCP exceeded 70%. XRD and XRF analysis results showed that the RWCP contained a large amount of Si, Ca and Al phase substances, which means that the RWCP has a potential for application in cementing materials.

### 3.2. Activity Index of RWCP

**Figure 4:**
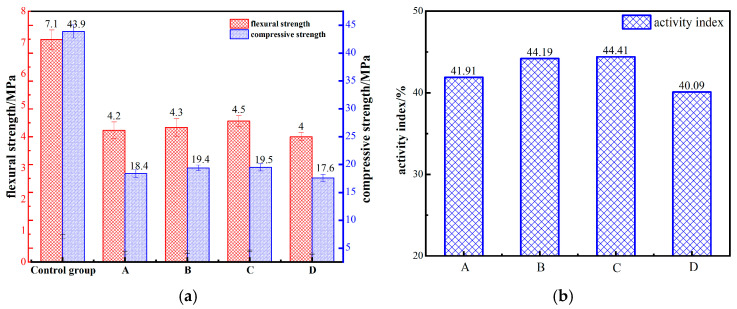
Bending strength, compressive strength of cement mortar test block containing RWCP, and activity index of RWCP. (**a**) Bending strength and compressive strength of cement; (**b**) Activity index of RWCP.

The activity of RWCP with different ball milling time was analyzed by a cement mortar test. The results of the cement mortar test and the activity analysis of RWCP are shown in Figure 4. The results show that there was no significant difference in the compressive strength and flexural strength of cement mortar specimens prepared by RWCP with different grinding times, and the activity index of recycled fine powder is similar to that of FA. When the mechanical activation time was 45 min, the activity index of the RWCP was up to 44.41% (Figure 4b). Therefore, the mechanical activation time of the RWCP used in the subsequent test was 45 min. With the further increase in activation time, the activity index of RWCP decreased to 40.09%, which means that there is an optimal mechanical strengthening time for RWCP, and excessive strengthening will lead to the decrease in activity of RWCP.

### 3.3. Strength Analysis of Concrete Containing RWCP

**Figure 5:**
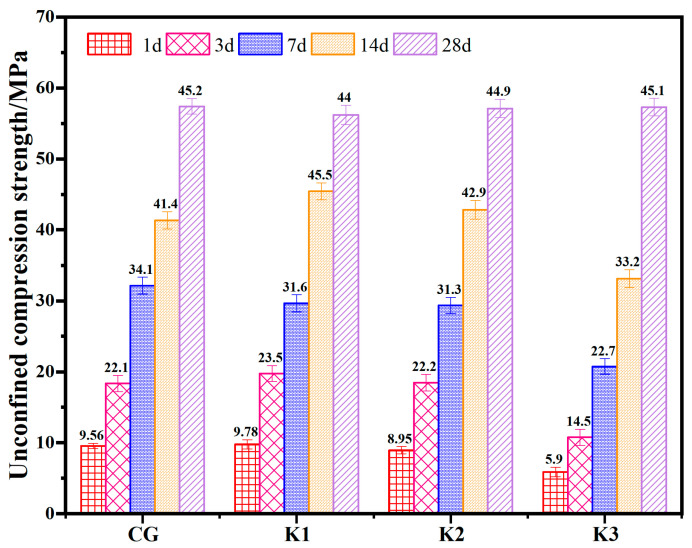
The UCS of the concrete test blocks with RWCP replacing BFS.

The proportion of the concrete control group adopts the proportion used in the actual production of C45 grade concrete of Qingdao Xinlong Building Materials Co., Ltd. from Qingdao, China. The replacement rate of RWCP for BFS in K1, K2 and K3 of the experimental group was 17.3%, 34.5% and 69.1%, respectively. The unconfined compressive strength (UCS) of the cube test block containing RWCP concrete after different curing ages is shown in Figure 5. The results showed that the incorporation of RWCP has no significant effect on the strength of concrete after curing 28 d. The UCS of K3 group showed that when the replacement rate of RWCP was 69.1%, the UCS of the concrete at 1, 3, 7, and 14 d decreased from 9.56, 22.1, 34.1, and 41.2 MPa to 5.9, 14.5, 22.7, and 33.2 MPa, respectively, which would be due to the low early activity index of RWCP. When the replacement rate of RWCP instead of BFS was 17.3%, the 3d strength increases from 9.56 MPa to 9.78 MPa, which may be due to the small average particle size of RWCP, which fills the gap of concrete and makes it have higher compactness. Therefore, in order to ensure the early and normal strength of concrete, the best replacement rate of RWCP instead of mineral powder should not be higher than 17.3%.

**Figure 6:**
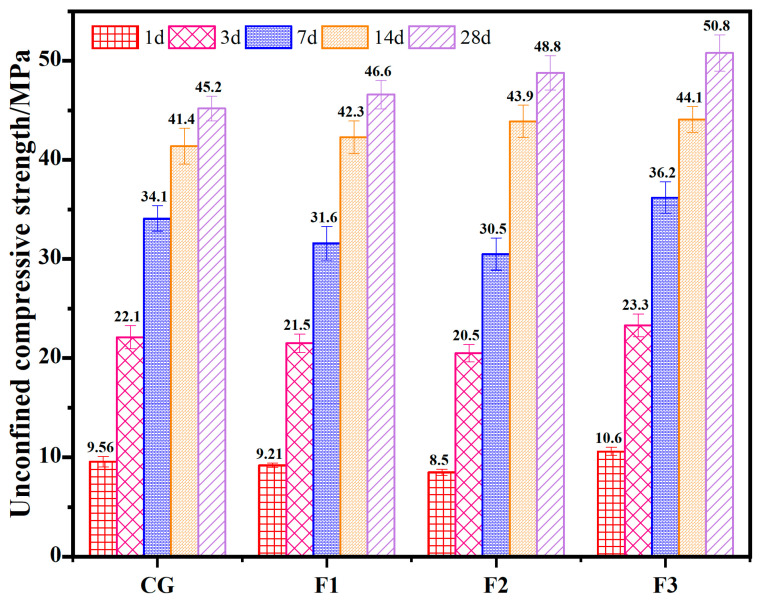
The UCS of the concrete test blocks with RWCP replacing FA.

Figure 6 shows the strength of the concrete prepared by RWCP instead of FA after different curing ages. The replacement rates of RWCP instead of FA in test groups F1, F2 and F3 are 25%, 50% and 100%, respectively. The results showed that the UCS of concrete in test groups K1, K2 and K3 after curing for 14 and 28 d is better than that in CG. This indicates that the use of RWCP instead of FA is beneficial to the strength development of concrete in the later stage. The UCS of concrete after curing 1, 3 and 7 d was lower than that of the CG when the replacement rate of FA replaced by RWCP was 25% and 50%, and the pre-stage and post-stage strength of concrete is better than that of the CG, when the replacement rate reaches 100%. When RWCP completely replaces FA in concrete, the 28-day strength of the concrete increases from 45.2 MPa to 50.8 MPa. The test results show that only considering the single factor of concrete compressive strength, RWCP can completely replace FA in the preparation of concrete.

### 3.4. Slump of Concrete Containing RWCP

**Figure 7:**
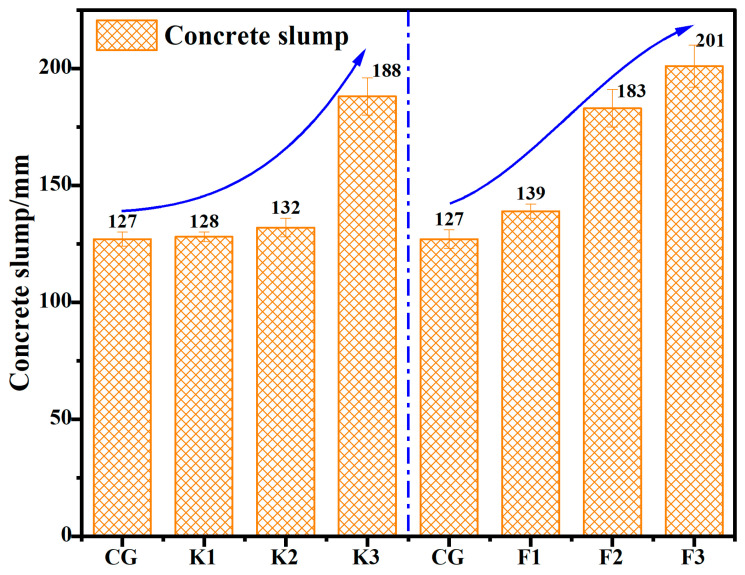
Slump of concrete containing RWCP.

The impact of replacement of BFS and FA with RWCP on the concrete slump is analyzed in Figure 7. The result indicate that the mixing of RWCP can prominently raise the slump and working property of concrete. When the replacement rate of BFS with RWCP hit 69.1%, the concrete slump was 188 mm. When FA was entirely replaced by RWCP, the slump of concrete was 201 mm. This could be explained by arguing that the RWCP through mechanical enhancement has changed the original porous structure and reduced the water absorption of RWCP. Additionally, as the grain size of RWCP is smaller than that of the raw material in concrete, the “balling effect” has enhanced the fluidity of concrete.

### 3.5. The Initial and Final Setting Time of Concrete Containing RWCP

**Figure 8:**
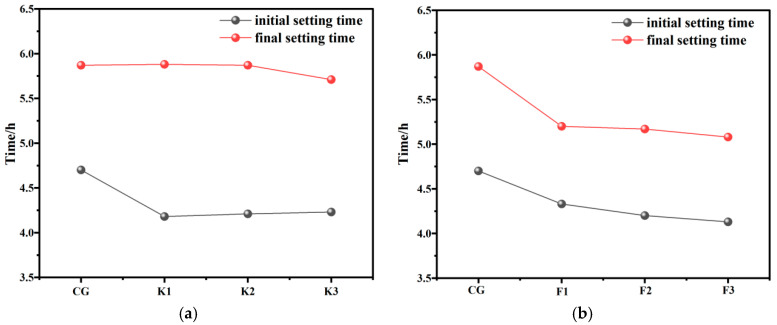
Analysis of initial and final setting time of concrete containing RWCP. (**a**) Analysis of initial and final setting time of concrete in which BFS is replaced by RWCP; (**b**) Analysis of initial and final setting time of concrete in which FA is replaced by RWCP.

The initial and final setting time of concrete containing RWCP is illustrated in Figure 8. The result shows that the initial setting time prominently decreases and the final setting time basically stabilizes when RWCP replaces BFS in concrete preparation. When the amount of admixture of RWCP hits 17.3%, the initial setting time reduces from 4.7 h to 4.18 h. When the replacement rate of BFS with RWCP further improves, the initial setting time of concrete mortar basically stabilizes at about 4.2 h. When RWCP replaces FA in concrete preparation, both the initial setting time and the final setting time decrease prominently. Moreover, as the replacement rate increases, the initial setting time and the final setting time are both shorter. It means that RWCP can effectively accelerate the cement hydration and are potentially useful in preparing early-strong paste injection materials. The result of XRD analysis indicate that RWCP contains some unhydrated C_3_A. When RWCP replaced BFS and FA, the C_3_A content in the concrete material system was higher than that in the CG, which helped accelerate the hydration of cementing material [30].

### 3.6. Impermeability of Concrete

In the high-salt environment, a large number of ions will penetrate into the concrete and corrode the steel structure through an electrochemical reaction, which will shorten the service life of reinforced concrete and even cause the problem of concrete cover shedding, which will have a negative impact on the quality and safety of reinforced concrete engineering [31,32]. The chloride ion migration coefficient and electric flux of concrete are important indexes to evaluate the impermeability of concrete.

#### 3.6.1. Electric Flux of Concrete

The electric flux test results of concrete are shown in Table 3. The results show that the electric flux of concrete increases with the increase in the amount of RWCP replacing BFS in concrete, which means the substitution of RWCP for BFS will lead to a decrease in the impermeability of concrete. The activity of RWCP is lower than that of BFS. The substitution of RWCP for BFS will reduce the degree of hydration and the amount of hydration products of concrete, which will lead to a decrease in the compactness and impermeability of concrete. The electric flux of concrete increases first and then decreases with the increase in the amount of RWCP mixed while using RWCP instead of FA. The electric flux of F2 concrete is the lowest, only 3483 C, and the impermeability grade reaches Q-II when evaluated by *Durability Test and Evaluation Standard of Concrete (JGJ/T 193-2009)* [33]. The average particle size of RWCP is smaller than that of cement and BFS, and higher than that of FA. The addition of appropriate amounts of RWCP is beneficial to improve the gradation of concrete, increase the compactness of concrete and improve the impermeability of concrete. When RWCP completely replaces FA in concrete, although the activity of RWCP is higher than that of FA, the strength performance of concrete is better, but the excessive replacement of FA by RWCP leads to poor gradation, thus reducing the impermeability of concrete. Therefore, in some projects with high impermeability requirements, the replacement amount of RWCP instead of FA should be strictly controlled.

#### 3.6.2. RCM Test

**Figure 9:**
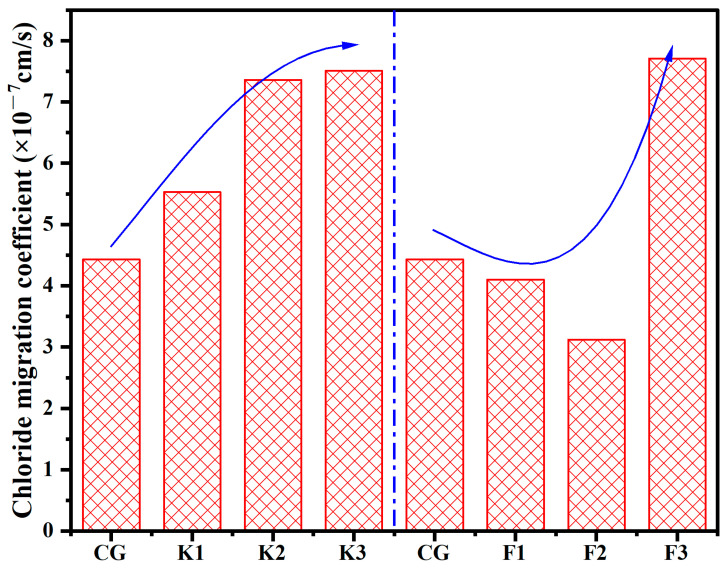
Chloride ion migration coefficient of concrete containing recycled concrete fines.

Figure 9 shows the chloride ion migration coefficient of concrete containing RWCP. The result show that the RCM test results were consistent with the electric flux test results. The chloride ion migration coefficient of F2 was the lowest, which was 3.12 × 10^−7^ cm/s, while the chloride ion migration coefficient of F3 was the highest, which was 7.71 × 10^−7^ cm/s. Therefore, in a high-salt environment, the content of RWCP in concrete should be strictly controlled, and excessive addition may lead to a decrease in the impermeability of concrete.

### 3.7. XRD Analysis

**Figure 10:**
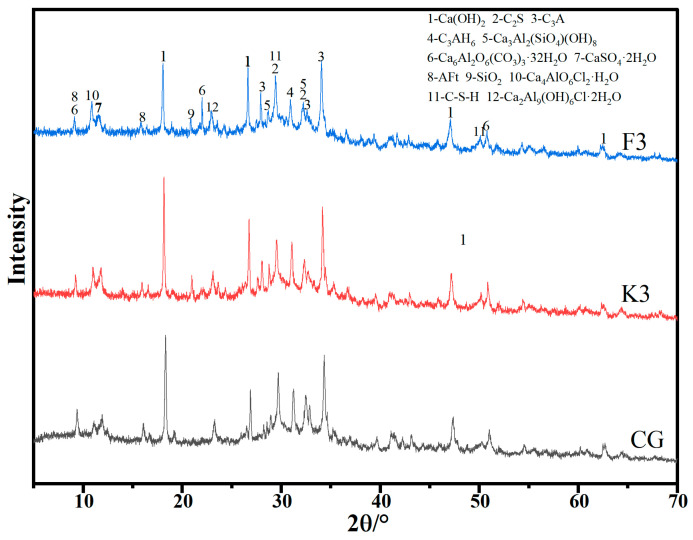
XRD diffraction patterns of CG, K3 and F3.

Figure 10 shows the XRD diffraction patterns of the CG, K3 and F3 paste test blocks cured for 28 d. The results showed that the hydration products in the three groups were basically the same, namely C_2_S, C_3_A, C_3_AH_6_, Ca_3_Al_2_(SiO_4_)(OH)_8_, AFt, and C-S-H, etc. These products were cemented with each other and connected the surrounding particles to improve the strength of concrete. This suggests that the primary role of RWCPs within the concrete paste is to enhance performance by filling and nucleating, thereby promoting the formation of hydration products in the matrix. The SiO_2_ composition in the aggregate of WC results in the characteristic peak strength of SiO_2_ in K3 and F3 being significantly higher than that in the CG. The wrapped peaks observed between 25° and 35°, as well as around 50°, are predominantly composed of C-S-H gel. From the dispersion peak observed at approximately 50°, it is evident that the addition of RWCP promotes the generation of more C-S-H in samples F3 and K3, which contributes to the enhancement of concrete strength. The XRD pattern indicates that the addition of RWCP results in the emergence of a new hydration product, Ca_6_Al_2_O_6_(CO_3_)_3_·32H_2_O, characterized by distinct peaks in F3 and K3. This formation is attributed to the carbonization of calcium aluminate present in the cement, suggesting that incorporating RWCP may enhance the carbonization process within the concrete. Furthermore, the characteristic peak intensities of Ca_4_AlO_6_Cl_2_·H_2_O and Ca_2_Al_9_(OH)_6_Cl·2H_2_O are greater than those in the CG, indicating an increased production of these substances. This increase is likely due to the introduction of chlorides from admixtures, such as antifreeze, which react with the calcium aluminate in the cement.

### 3.8. SEM Analysis

**Figure 11:**
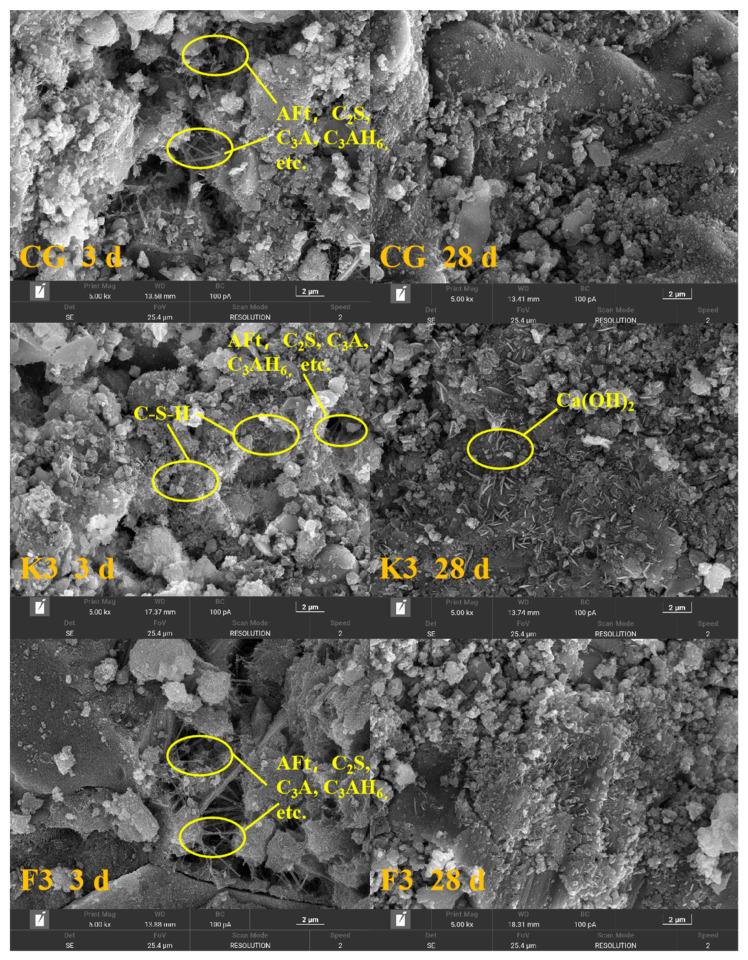
SEM analysis diagram of CG, K3 and F3 after curing for 3 d and 28 d.

Figure 11 is the electron micrograph of the CG, K3 and F3 paste test blocks cured for 3 d and 28 d. The results show that there are significant needle-like hydration products in the electron micrograph of the CG and F3 after curing for 3 d, while there are no obvious hydration products in the K3 specimen. The UCS test results also show that the strength of the K3 group was significantly lower than that of the CG and F3, which was consistent with the results of the electron microscope analysis. This is because RWCP replaces BFS in concrete, a highly active mineral admixture, resulting in a lower degree of early hydration. After curing for 28 d, the structural compactness of the CG, K3 and F3 paste test blocks was significantly improved, and the improvement of structural compactness will enhance the impermeability of the concrete system. After curing for 28 d, there were flake products on the surface of K3 group specimens. Combined with the results of XRD analysis, it was considered that the flake products were Ca(OH)_2_, and the formation of Ca(OH)_2_ would lead to expansion, which caused microcracks in concrete, thus affecting the compressive strength and impermeability of concrete. The needle-like products include AFt, C_2_S, C_3_A, C_3_AH_6_, and zeolite phases, while the gel products consist of C-S-H; these products contribute positively to the development of concrete strength. The results of the RCM test also show that the chloride ion migration coefficient of concrete increases with the increase in the substitution rate of RWCP instead of BFS, which means that the substitution of RWCP for BFS will lead to a decrease in impermeability of concrete.

## 4. Conclusions

In this paper, RWCP was prepared from WC after mechanical strengthening and used to replace BFS and FA in the preparation of concrete. The particle size distribution, chemical properties and activity index of RWCP were analyzed, and the effects of RWCP on the mechanical properties, working properties and impermeability of concrete were investigated. The main conclusions are as follows:

(1) The fineness of recycled fine powder particles of WC decreases with the increase in mechanical strengthening time, which is mainly composed of quartz, gismondine, C_2_S, cancrinite and portlandite. After mechanical activation of WC for 45 min, the best activity index is 44.41%.
(2) The use of RWCP instead of BFS for the preparation of concrete will lead to a decrease in the permeability of concrete. The content of RWCP instead of BFS for the preparation of concrete should be lower than 17.3%. When the substitution amount is less than 17.3%, the early and middle age strength of concrete can be improved. When the substitution rate of RWCP instead of BFS is greater than 17.3%, the early and middle strength of concrete will decrease. When BFS replaces FA for the preparation of concrete, the strength of concrete increases with the increase in replacement rate. When the replacement rate of RWCP replacing FA was 66.6%, the concrete had the best impermeability.
(3) When the RWCP is used to replace the BFS and FA for the preparation of concrete, the slump of concrete can be improved, which is beneficial to the fluidity of concrete. The RWCP has some unhydrated C_3_A, which promotes the hydration process and reduces the initial setting time of concrete.
(4) The hydration products of RWCP concrete containing RWCP were basically consistent with the CG. The main hydration products were C_2_S, C_3_A, C_3_AH_6_, Ca_3_Al_2_(SiO_4_)(OH)_8_, CaAlO(CO_3_)_3_·32H_2_O and AFt,, etc. These hydration products are interlaced and fill the pores to improve the strength and compactness of concrete.

## Figures and Tables

**Table 1:** Concrete test ratio (kg/cm^3^).

Group	Sand	Stone	Cement	RWCP	BFS	FA	Water	Water Reducer
Control group (CG)	930	812	314	/	52.1	36	140	11.25
K1	930	812	314	9	43.1	36	140	11.25
K2	930	812	314	18	34.1	36	140	11.25
K3	930	812	314	36	16.1	36	140	11.25
F1	930	812	314	9	52.1	27	140	11.25
F2	930	812	314	18	52.1	18	140	11.25
F3	930	812	314	36	52.1	0	140	11.25

**Table 2:** Analysis of chemical compounds in RWCP.

Chemical Element Analysis/%											
Constituents	Na_2_O	MgO	Al_2_O_3_	SiO_2_	P_2_O_5_	SO_3_	K_2_O	CaO	TiO_2_	Fe_2_O_3_	LOI
wt.%	2.817	1.735	12.998	52.46	0.164	0.723	3.722	11.436	0.429	3.077	10.1

**Table 3:** Electric flux of concrete.

Group	Electric Flux/C	Grade
CG	3763.44	Q-II
K1	3964.68	Q-II
K2	4333.68	Q-I
K3	4363.12	Q-I
F1	3612.24	Q-II
F2	3483	Q-II
F3	5659.704	Q-I

## Data Availability

The original contributions presented in the study are included in the article, further inquiries can be directed to the corresponding author.
